# The influence of avoidant attachment and perceived support on disclosure about involvement in donor-assisted conception to family and friends

**DOI:** 10.1093/humrep/dead019

**Published:** 2023-02-03

**Authors:** Iolanda S Rodino, Katherine A Sanders

**Affiliations:** Medical School, The University of Western Australia, Perth, Western Australia, Australia; School of Human Sciences, The University of Western Australia, Perth, Western Australia, Australia

**Keywords:** attachment, counselling, disclosure, donor-assisted conception, support, third party reproduction

## Abstract

**STUDY QUESTION:**

Do the attachment-related dimensions *Anxiety* and *Avoidance* and perceived partner and social support in recipients and donors influence disclosure to others about their involvement in donor-assisted conception (DAC)?

**SUMMARY ANSWER:**

A higher global score on attachment Avoidance was associated with greater non-disclosure about involvement in DAC by participants to relationship-specific others.

**WHAT IS KNOWN ALREADY:**

Within the context of DAC, the topic of disclosure has been investigated in terms of the ‘if’, ‘when’, and the ‘how’ to disclose about circumstances of conception. Less focus, however, has been directed to investigating psychological theoretical frameworks that influence disclosure decisions to others, i.e. to whom information is disclosed and to what extent details are transparently revealed about the donor programme.

**STUDY DESIGN, SIZE, DURATION:**

The study was of a cross-sectional design and utilized a sample of 301 participants who were, or had been, involved in DAC, and were recruited across states of Australia. An online self-report questionnaire was completed between June 2014 and June 2017.

**PARTICIPANTS/MATERIALS, SETTING, METHODS:**

English speaking participants consisting of 209 female recipients and 92 donors (36 sperm; 48 egg; 8 embryo donors). Of the recipients, 104 had successfully conceived children via donated gametes (68 sperm, 23 eggs, 6 embryos, and 7 recipients where both gametes were donated from 2 donors to create the embryo). Participants anonymously completed an online questionnaire consisting of five sections: Demographics, Donor Conception and Disclosure Practices, the Experiences in Close Relationships-Relationships Structure, the Quality of Relationship Inventory, and the Multidimensional Scale of Perceived Social Support. Pearson correlations, independent samples *t*-tests, Chi-square, and ANOVA were used to explore the association between attachment *Anxiety* and *Avoidance* scores and disclosure about involvement in a DAC programme to significant others (i.e. parents, siblings, in-laws, and friends).

**MAIN RESULTS AND THE ROLE OF CHANCE:**

Compared to published community cohort data, participants reported lower global scores on attachment *Anxiety* and *Avoidance* and high levels of romantic partner and social network support, suggestive of secure relationships in the overall study sample. A higher score on attachment *Avoidance* was associated with less disclosure to significant others in their social network (i.e. parents, siblings, in-laws, and close friends), even in the presence of strong partner support (partial *r* = −0.248, *P* = 0.005). Higher scores on attachment *Avoidance* were inversely associated with level of perceived partner and social network support (all *P* < 0.05). Irrespective of attachment scores, more than 90% of all participants agreed that a child born of DAC should be told about mode of conception.

**LIMITATIONS, REASONS FOR CAUTION:**

This study utilized a cross-sectional design precluding causal inferences between dimensions of insecure attachment and disclosure practices. Participants were required to self-report on the quality of their relationships with the potential for social desirability respondent bias. The study’s self-selecting sample may limit generalization to participants who were dis-inclined to participate. Specifically, respondents who have an *Avoidant* attachment style, may have elected not to participate in the study.

**WIDER IMPLICATIONS OF THE FINDINGS:**

Given the increased use of biotechnology and digital facial recognition enabling self-discovery of the donor and the donor’s extended family, non-disclosure about involvement in DAC may have consequences. An *‘Avoidant’* attachment style is important to assess as a potential risk factor for non-disclosure about involvement in DAC across different relational contexts (e.g. close family members and friends). Fertility counsellors should consider introducing a measure of attachment screening as a pre-emptive psychoeducational strategy during donor implications counselling. This information could be used to offer patients insight into concerns they have about DAC disclosures to key important relationships, providing a target of clinical intervention.

**STUDY FUNDING/COMPETING INTEREST(S):**

No external funds were sought for this work. None of the authors have any competing interests to declare.

**TRIAL REGISTRATION NUMBER:**

N/A.

## Introduction

For many individuals and couples becoming a parent is a pivotal life moment. Consequently, the inability to conceive children can be experienced as a major source of subjective and interpersonal distress (Rooney and Domar, 2018; Wischmann and Thorn, 2022). This distress can be magnified in fertility treatment programmes involving donor-assisted conception (DAC), where reliance upon the assistance of another party is required but may not be the first preference, nor an option supported by significant others. Challenges to adjustment relating to DAC can continue during the transition to parenthood. These can surface when parents are faced with disclosure decisions to the donor conceived person, family and social networks, warranting psychoeducation, and support to ensure donor conceived individual’s rights to donor information are given due consideration (Tallandini *et al.*, 2016; Crawshaw and Daniels, 2019; Ishii and De Miguel Beriain, 2022).

Historically, infertility and ART, particularly treatment involving the use of donated gametes, was not widely discussed beyond the recipient couple and an atmosphere of stigma and secrecy prevailed (Daniels and Taylor, 1993; Readings *et al.*, 2011; Frith *et al.*, 2018). Social attitudes towards the use of donor gametes/embryos, however, have evolved recognizing the identity needs for donor-conceived people with increased transparency about a child’s genetic heritage and endorsement of open-identity donors (Scheib *et al.*, 2003; Scheib *et al.*, 2005; Van den Akker, 2006; Jadva *et al.*, 2009; Rodino *et al.*, 2011; Allan, 2012; National Health Medical Research Council (NHMRC), 2017; Dempsey *et al.*, 2019; Ishii and De Miguel Beriain, 2022). Moreover, new techniques to analyse DNA sequences and digital facial technology have allowed tracing of genealogies enabling self-discovery of the donor. These scientific advances have meant that absolute secrecy about DAC and donor anonymity can no longer be guaranteed, with implications for some, being the need for counselling (Harper *et al.*, 2016; Zadeh, 2016; Crawshaw, 2018; Crawshaw and Daniels, 2019; Pennings, 2019; Ishii and De Miguel Beriain, 2022; Łukasiewicz and Allan, 2022).

With societal, legislative, and biotechnology changes leaning towards openness, it is reasoned that being able to talk about the implications of DAC to significant others is an important task of the adaptation process for individuals/couples contemplating DAC treatment. This is irrespective of whether one is donating or receiving gametes/embryos. Specifically, transparency about involvement in DAC can have important interpersonal ramifications not only for the donor, recipient, and resulting child(ren) (Paul and Berger, 2007; Shehab *et al.*, 2008; Daniels *et al.*, 2011; Frith *et al.*, 2018), but also for family members of the donor who may view genetic lineage as important and therefore feel that they too have the right to know if a child that is genetically related to them was born into another family (Beeson *et al.*, 2013; Burke *et al.*, 2021). Nevertheless, for some individuals the revelation about one’s infertility (Slade *et al.*, 2007) or use of donor gametes/embryos may not be an easy process to emotionally navigate, impeding disclosure practices. Therefore, factors that potentially influence disclosure about DAC are important to explore from the perspective of all stakeholders.

To date, research investigating the issue of disclosure practices has predominantly focused on variables that influence disclosure decisions by recipient parents to their child(ren) and disclosure outcomes. These in part include parent–child relationship and communication issues, child emotional adjustment, parental psychological adjustment, impact of secrets, avoidance of stigma, identity needs, concerns about inadvertent consanguinity, and cultural and religious issues (Brewaeys *et al.*, 1997; Nachtigall *et al.*, 1997; Scheib *et al.*, 2003; Shehab *et al.*, 2008; Jadva *et al.*, 2009; Daniels *et al.*, 2011; Jadva *et al.*, 2011; Freeman and Golombok, 2012; Blake *et al.*, 2014; Rueter *et al.*, 2016; Tallandini *et al.*, 2016; Zadeh *et al.*, 2017). Relatedly, less common in DAC research is the exploration of the extent of disclosure about treatment parameters to others within the familial and/or social network. Söderström-Anttila *et al.* (2010) report that many couples undertaking routine IVF treatment selectively disclosed information to some of their social network about their IVF treatment, however withheld the same information from others. Jadva *et al.* (2011) and similarly Readings *et al.* (2011) discuss the notion of partial disclosure, whereby recipients inform their donor conceived offspring and/or relatives of their use of fertility treatment (e.g. that the child is an IVF baby), but in their communication are not transparent about their use of donated gametes. Consequently, individuals can vary considerably in their sharing of personal information with others dependent upon who that person is, and perhaps how secure/insecure they feel within that specific relationship. We propose that attachment theory, a theory about interpersonal relationships, support seeking and emotional regulation may, in part, provide insight as to why recipients and donors elect to selectively disclose about their involvement in DAC to significant others (Bowlby, 1969; Ainsworth *et al.*, 1978; Main, 1985; Bowlby, 1988; Mikulincer *et al.*, 2003; Shaver and Mikulincer, 2007).

Attachment theory (Bowlby, 1969; Ainsworth *et al.*, 1978; Main, 1985; Bowlby, 1988) pertains to a psychological model that fundamentally explores human relationships, particularly close relationships. Bowlby (1969) first hypothesized ‘attachment’ as originating from early life interactions with at least one pivotal caregiver that served to influence later affectional bonds across the lifespan. In adulthood, this includes perceived availability of another person as a secure and safe haven, especially when encountering challenging life events and the perceived responsiveness of that person to assist with emotional regulation of such challenges (Shaver and Mikulincer, 2007). Generally, with respect to adult relationships and social support, three primary attachment styles have been conceptualized: secure and two forms of insecure dimensions comprising anxious and avoidant styles (Ainsworth *et al.*, 1978; Bowlby, 1988; Brennan *et al.*, 1998; Fraley *et al.*, 2011; Mikulincer and Shaver, 2016). Of the insecure dimensions, people high in attachment-related avoidance are overly self-reliant and may dismiss the importance of relationships. They have more mistrusting views of the world and thus may be less comfortable about self-disclosures. In contrast, those high in attachment-related anxiety may disclose information too readily. They may require excessive re-assurance about life choices or have enhanced perceptions of threat and rejection. Secure attachment is a balance of lower levels of both insecure dimensions (Mikulincer and Nachshon, 1991; Mikulincer and Florian, 1995; Brennan *et al.*, 1998; Fraley *et al.*, 2011) and is associated with greater relationship satisfaction and increased interpersonal closeness. Moreover, it is associated with greater efforts of giving, seeking, and utilizing emotional support, and fewer fears about negative reactions (Hazan and Shaver, 1987; Simpson, 1990; Mikulincer and Shaver, 2016).

An individual’s attachment style has been viewed as an enduring trait influencing expectations and experiences of people generally across the lifespan including romantic partner support (Ainsworth *et al.*, 1978; Hazan and Shaver, 1987; Fraley and Shaver, 2000). Fraley *et al.* (2011), however, propose that individuals develop attachment presentations that are relationship specific rather than unilaterally applicable to every relationship formed. As such, it is possible to consider that attachment-related insecurities and perceived support may differentially shape disclosure decisions an adult makes with their family of origin and significant others, such as partner or close friends.

Studies that explore attachment theory in the context of fertility treatment and/or post-fertility treatment are limited although broadly suggest that attachment insecurities are inversely associated with relationship satisfaction, perceived support and/or socio-emotional adjustment (Mikulincer *et al.*, 1998; Gibson *et al.*, 2000; McMahon and Gibson, 2002; Bayley *et al.*, 2009; Mahajan *et al.*, 2009; Zadeh *et al.*, 2017; Purcell-Levesque *et al.*, 2019; Elyasi *et al.*, 2021). To the authors’ knowledge, no study to date has specifically explored the concept of attachment, nor its relationship with perceived support, as variables associated with DAC disclosure, representing a gap in the empirical literature.

The overarching aim of this study was to explore in recipients and donors the association between attachment style and their disclosure of their involvement in DAC to significant others. Since experiences within relationships can trigger attachment insecurities (Mikulincer *et al.*, 2003; Fraley *et al.*, 2011; Mikulincer and Shaver, 2016) recipient and donor attachment style was further explored in the context of perceived quality of romantic partner relationship and social network support. From a clinical perspective, research exploring attachment style may inform discussions within pre-treatment implications counselling that provide a safe base for discussing barriers to disclosure in recipients and donors contemplating DAC.

## Materials and methods

### Participants

This study involved 301 participants, comprising 209 female recipients and 92 donors (36 sperm; 48 egg; 8 embryo donors). Of recipients, 104 had successfully conceived children via donated gametes (68 sperm, 23 eggs, 6 embryos, and 7 recipients whereby both gametes were separately donated to form the embryo). Of the 104 recipients with children, 8 had conceived with the assistance of a friend, 18 via a known donor recruited through social media advertising, and 78 used a donor recruited by their treating clinic, and whom remained unknown to them. Of donors, 46.7% were raising children. All participants were adults 18 years or older and were conversant in English.

### Questionnaire composition

Two similarly structured versions of the study questionnaire were used to gauge the attitudes of recipients and donors. Each questionnaire included five main sections: Demographics; Information about the type of donor programme (all participants) and donor’s motivations (donor only); Participant attitudes to disclosure and actual disclosure to others in their familial and social network, specifically children born of DAC, mother, father, siblings, mother-in-law, father-in-law, partner’s siblings, closest friend, partner’s closest friend; Participant experiences of close relationships with family members and friends, as gauged by the Experiences in Close Relationships-Relationships Structure Questionnaire (ECR-RS) (Fraley *et al.*, 2011); and responses to two support measures: Quality of Relationship Inventory (QRI) (Pierce *et al.*, 1997) and the Multidimensional Scale of Perceived Social Support (MSPSS) (Zimet *et al.*, 1988). Participants were not required to answer all questionnaire sections, logistically being determined by donor programme type, relationship status, and presence or absence of children.

The ECR-RS (Fraley *et al.*, 2011) is a 36-item self-report questionnaire designed to assess attachment patterns with four specific close relationship domains (i.e. mother, father, partner, and close friend). The ECR-RS measures two dimensions of insecure attachment to significant others, namely *Avoidance* (six items) and *Anxiety* (three items) for each domain. Items are scored on a 7-point Likert scale, from strongly disagree to strongly agree, with higher scores indicating greater attachment avoidance and attachment anxiety with that relationship type. Scores were calculated according to Fraley *et al.* (2011). To calculate *Global Anxiety* or *Global Avoidance*, the scores of relationship specific attachments (mother, father, partner, and close friend) were averaged, being adjusted to take into account the capacity for the participant to disclose to the nominated relationship (e.g. when a parent was deceased). The ECR-RS possesses high internal consistency with Cronbach’s alpha of 0.85 and 0.88, respectively, for global insecure attachment factors *Anxiety* and *Avoidance* (Fraley *et al.*, 2011). In the current study Cronbach’s alpha for attachment *Anxiety* and *Avoidance* factors were, respectively, 0.79 and 0.91 for recipients and 0.81 and 0.89 for donors, demonstrating comparable internal consistency.

The QRI (Pierce *et al.*, 1997) is a widely used measure of support. The scale consists of 25 items, which is used to measure Support, Conflict, and Depth of a current relationship with a romantic partner. Items are scored on a 4-point Likert scale, with a score of 1 reflective of the item response ‘not at all’ and 4 being ‘very much’. Only the Support subscale (seven items), which measures the individual’s perception of their partner to be a source of assistance, was used in the current study. The *QRI-Support* subscale possesses high internal consistency and shows good reliability, with a Cronbach’s alpha >0.83 (Pierce *et al.*, 1997). For study participants, the Cronbach’s alpha for the QRI was 0.80 for recipients and for donors 0.88.

The MSPSS was used to evaluate beliefs about broader perceived social support (Zimet *et al.*, 1988). The scale assesses perceptions about support from family, friends, and significant others. The scale consists of 12 questions with items rated on a 6-point Likert scale ranging from 1 to 6, with participants rating the extent they agree with the item. A global score is generated from the average of the three subscales. The MSPSS possesses high internal consistency and shows good reliability, with a Cronbach’s alpha >0.83 (Zimet *et al.*, 1988). In this study, Cronbach’s alpha was comparable, with respective scores of 0.90 for the recipients and 0.92 for donors.

### Procedure

Information about the online study was posted on websites hosted by donor conception support groups, state reproductive technology councils, the Australian and New Zealand Infertility Counsellors’ Association (ANZICA), and interested fertility clinics. Following informed consent, individuals who were participating in, or had previously participated in, a donor conception fertility treatment programme in Australia anonymously completed an online questionnaire. Data were collected between June 2014 and June 2017. Questionnaire completion took approximately 20 minutes and participants could withdraw at any stage prior to endorsing final submission. Ethics approval for the study was obtained from The University of Western Australia (Reference Number: RA/4/1/5249).

### Data analysis

Descriptive statistics were used to summarize sociodemographic and psychosocial responses for each participant group (i.e. recipient and donors) and, where indicated, donor programme subgroup (i.e. donor sperm recipients, donor egg recipients, donor embryo recipients; egg, sperm, and embryo donors). *Global Anxiety* and *Global Avoidance* scores were analysed according to donor programme and relationship-specific disclosures patterns. Independent samples *t*-tests were used to compare recipients and donors on the ECR-RS attachment dimensions, QRI, and MSPSS scores. One sample *t*-tests were used to compare *Global Anxiety* and *Global Avoidance* scores to the means of the Fraley *et al.* (2011) normative sample. Correlational analyses were conducted to analyse the association between attachment scores and the extent of social network disclosure. SPSS for Windows, Version 26 (SPSS, Chicago, IL, USA) was used for all statistical analyses and a *P*-value of  < 0.05 was considered to indicate statistical significance.

## Results

### Sample characteristics

#### Recipients

Sociodemographic characteristics of the respondents are shown in Table I. A total of 209 recipients were involved in this study (mean age = 39.9 years; SD = 7.4). On an average, recipients with children (n = 104) were 42.3 years of age (SD = 8.05) and those without children (n = 105) were 37.5 years (SD = 5.77). Of those with children, the majority (75%) had conceived via an Australian fertility clinic; the remainder had conceived through overseas clinic fertility arrangements. The majority of donor conceived children were aged  < 10 years (range 0–40 years; median = 3 years). Of recipients (n = 105) still trying to conceive, few (n = 12) were seeking overseas treatment.

**Table I:** Sociodemographic and disclosure characteristics of respondents in a study of disclosure about involvement in donor-assisted conception.

Characteristic	Recipient (n* *=* *209) %	Donors (n* *=* *92) %
Sex		
Female	100	61
Male	0	39
Relationship type		
Heterosexual	63.4	Not asked
Same sex	35.8	Not asked
Current relationship status		
Married/defacto	58.4	64.1
Dating	3.8	13.0
Single	37.8	22.8
Involved in parenting children		
Yes	49.8	46.7
No	50.2	53.3
Treatment programme type		
Egg	27.6*	52
Sperm	64.3*	39
Embryo	3.3*	9
Double gamete	3.8*	NA
^#^Disclosure practices		
Disclosed to no-one	3.8	5.4
Partial network disclosure	60.3	75
Full network disclosure (disclosure to anyone)	35.9	19.6
Relationship-specific disclosure		
Mother	91.8	81.5
Father	85.2	76.5
Siblings	86.1	78.0
Mother-in-law	72.8	62.3
Father-in-law	69.8	50.9
Partner’s siblings	73.4	52.4
Closest friend	95.0	94.4
Partner’s friend	87.0	64.8

NA, not applicable.

* Respondents on Item n* *=* *185; 24 recipients did not respond.

# Refers to disclosure about donor-assisted conception to social network, adjusted for absent relationships.

#### Donors

Of 92 donors, 48 were egg donors, 36 were sperm donors, and 8 participants donated embryos (mean age = 39.54 years; SD = 12.10). The majority were currently donating (57%) whilst the remainder had donated prior to the start of study recruitment. Most donors were heterosexual partnered (69.6%). Forty-four donors (33 sperm, 7 egg, and 4 embryo) indicated they were recruited by a clinic while 8 egg donors were recruited by family and/or friend acquaintances. Forty donors were recipient recruited via advertisement (3 sperm, 33 egg, and 4 embryo).

### Disclosure patterns

#### Recipients

Overall, a majority of recipients (94%) agreed that a child born of DAC should be told about the manner of their conception. Of recipients with children (n = 104), 73% had disclosed to their donor conceived offspring about the mode of their conception whilst the remainder had not disclosed, primarily owing to the age of the child. Amongst recipients, distribution of disclosure patterns to significant others varied (Table I); 35.9% indicated they had been open with all nominated relationships about their involvement in DAC (full social network disclosure), 60.3% had informed some of their social network but not others, while 8 recipients (3.8%) had disclosed to no-one about their involvement in DAC (complete non-disclosure) (Table I). Proportionally more single women (48%) disclosed to their available social network compared to a partnered heterosexual recipient (24.7%) and same sex recipients (37.5%) (χ^2^_(df = 2)_ = 9.433, *P* = 0.008).

#### Donors

A total of 46.7% were involved in the parenting of children (Table I). Of donors the majority (91.3%) agreed or strongly agreed that a child born of DAC should be informed about the manner of their conception. The remaining were neutral (four female, four male). Of those donors with children (n = 55), the majority (90%) had disclosed to child(ren) they were currently parenting about their involvement in DAC. All partnered donors had disclosed to their current partner. Approximately 19.6% of donors indicated that they had fully disclosed about their involvement in DAC to their social networks (i.e. full disclosure). A further 75% of donors indicated they had informed some individuals in their social network but not others (i.e. partial disclosure) and 5.4% of donors had disclosed to no-one. Proportionally more donors had disclosed their involvement in DAC to close friends than to their parents (Table I).

### Relation between attachment dimensions and disclosure

#### Recipients

As a group, recipients experienced significantly lower global *Anxiety* and *Avoidance* attachment scores compared to the Fraley *et al.* (2011) community female reference group (*Anxiety*: *t*(208) = −12.67 *P* < 0.001; *Avoidance*: *t*(208) = −6.05, *P* < 0.001) (Table II). Recipients who were single exhibited a higher *Avoidance* score (*M = *2.92, SD = 1.06) than married recipients (*M* = 2.59, SD = 0.99) (*t* = 2.27; *P* = 0.024); there was no significant difference on attachment *Anxiety* score (*P* > 0.05). For recipients who were single, extent of full disclosure about DAC to significant others was negatively associated with attachment score (*Anxiety*: *r* = −0.419; *P < *0.001; *Avoidance*: *r* = −0.301; *P* < 0.001). For recipients who were partnered, extent of disclosure about DAC was also negatively associated with *Avoidance* (*r* = −0.300; *P* < 0.001) but not *Anxiety* attachment score (*r* = −0.120; *P = *0.166). Accounting for the presence or absence of children did not change the relation between attachment scores and extent of disclosure for single (*Avoidance* partial *r* = −0.307, *P* = 0.008; *Anxiety r* = −0.428, *P* < 0.001) or partnered women (*Avoidance* partial *r* = −0.308, *P* < 0.001; *Anxiety* partial *r* = −0.132, *P* = 0.130).

**Table II:** Participant scores according to psychological variables.

	Recipients (N = 209)	Donors (N = 92)		Fraley *et al.* (2011) cohort (N = 388)		Christian *et al.* (2019) cohort (N = 695)^#^
		Male N = 36	Female N = 56	Male N = 136	Female N = 252	
Anxiety attachment	1.80 (0.86)	1.89 (0.95)	1.90 (0.92)	2.42 (1.11)	2.56 (1.21)	3.57 (1.41)
	N = 209	N = 36	N = 56			
Avoidance attachment	2.71 (1.03)	2.84 (0.99)	2.71 (1.10)	3.34 (0.96)	3.14 (0.96)	3.61 (1.26)
	N = 209	N = 36	N = 56			
*QRI-support	3.75 (0.35)	3.60 (0.55)	3.78 (0.35)			
	N = 134	N = 27	N = 45			
Global MSPSS	58.10 (11.69)	58.25 (9.01)	60.34 (12.68)			
	N = 199	N = 36	N = 56			

Data are presented as mean (SD).

Multidimensional Scale of Perceived Social Support (MSPSS) global score is generated from the average of three subscales (friends, family, significant others); Quality of Relationships (QRI) score is generated from average scores on seven support items; Attachment dimensions are calculated from average scores on six *Avoidance* test items and three *Anxiety* test items across four relationship types (mother, father, partner, close friend).

* QRI applicable only for partnered participants.

# Christian *et al.* (2019) used a modified version of the Experiences in Close Relationships-Relationships Structure Questionnaire, which included three additional items for the anxiety subscale. Scores by gender not given; 53.5% of their sample is female.

Differential disclosure patterns to key parental figures were reported. For recipients, relationship specific attachment *Anxiety* score was not significantly associated with non-disclosure to their mother (Non-disclosure *M* = 1.64 (SD = 1.17); Disclosure: *M* = 1.51 (SD = 1.02), *P* > 0.05), however, those recipients who were not open with their mother had significantly higher relationship specific *Avoidance* scores (Non-disclosure: *M* = 4.84, (SD = 1.54); Disclosure: *M* = 2.68 (SD = 1.56), *P* < 0.001). For disclosure about DAC to fathers, those recipients who did not discuss use of donor treatment had significantly higher scores on both attachment *Anxiety* (Non-disclosure: *M* = 2.62 (SD = 1.65); Disclosure: *M* = 1.75 (SD = 1.20), *P* = 0.03) and *Avoidance* (Non-disclosure: *M* = 5.40 (SD = 1.51); Disclosure *M* = 3.34 (SD = 1.56), *P* < 0.001).

#### Donors

Male and female donors had significantly lower levels of global *Anxiety* and *Avoidance* attachment scores compared to the Fraley *et al.* (2011) male and female comparative reference groups (all *P* < 0.001) (Table II). Attachment *Anxiety* and *Avoidance* scores did not differ between male and female donors (Table II).

Among single donors there was no association between either attachment *Anxiety* or *Avoidance* scores and disclosure (*P* > 0.05). Among partnered donors, there was a trend towards higher *Avoidance* attachment and disclosure to fewer individuals in their network (*r* = −0.229, *P* = 0.055). With respect to disclosure patterns to key parental figures, differential patterns were reported. Donors who had not disclosed their involvement in DAC to their father reported significantly higher levels of attachment *Anxiety* with their father (*M* = 2.64, SD = 1.69) compared to those who had disclosed to their father (*M* = 1.64, (SD = 1.11); *P* = 0.004). Similarly, those who did not disclose to their father had higher levels of attachment *Avoidance* with their father (M = 4.04, SD = 1.64) compared to those who did disclose (*M* = 3.20, (SD = 1.62); *P* = 0.021). Whether or not donors had disclosed to their mother did not relate to level of relationship specific *Anxiety* or *Avoidance* attachment scores (all *P *>* *0.05).

### Relation between social support, disclosure, and attachment

Respondents with partners uniformly reported high levels of partner support on the QRI with no differences between recipients and donors. A non-significant trend for male donors to report slightly lower QRI score than female donors was observed (*P* = 0.09; Table II). There was no difference between recipients and donors in mean score of global MSPSS (Table II). Single women had lower global support scores on the MSPSS compared to partnered women (*t* = −5.38, *P* = 0.001).

Overall, for recipients and donors, a higher level of perceived support (QRI and MSPSS) was significantly associated with lower scores on attachment *Avoidance* (Table III). Higher support scores were also significantly associated with lower global *Anxiety*, with the exception of QRI support for partnered recipients and MSPSS for single donors (Table III). Participants who had disclosed had higher mean MSPSS global scores than those who did not (*P* = 0.034). Greater level of *Avoidance* attachment was associated with non-disclosure to significant others in their social network (i.e. parents, siblings, in-laws, and friends), even in situations of strong partner support (partial *r* = −0.248, *P* = 0.005).

**Table III:** Pearson’s correlations between relationship status, perceived support, and attachment factors among recipients and donors.

Donor programme	Support measure	Global avoidant score	Global anxious score
Recipients			
Single	QRI	N/A*	N/A
	MSPSS	−0.451^b^	−0.251^a^
Partnered	QRI	−0.327^b^	−0.161 (n.s.)
	MPSS	−0.682^b^	−0.471^b^
Donors			
Single	QRI	N/A*	N/A*
	MSPSS	−0.760^b^	−0.053 (n.s.)
Partnered	QRI	−0.473^b^	−0.369^c^
	MSPSS	−0.733^b^	−0.503^b^

QRI, quality of relationships; MSPSS, multidimensional scale of perceived social support; n.s., correlation not significant.

* N/A QRI test score not applicable for single recipients or donors.

a Correlation is significant at the 0.05 level (two-tailed).

b Correlation is significant at the 0.001 level (two-tailed).

c Correlation is significant at the 0.002 level (two-tailed).

## Discussion

For some, involvement in DAC can activate attachment needs which may have bearing upon disclosure decisions to the donor conceived person, family, and significant others. In this study, attachment theory (Bowlby, 1969, 1988) forms the theoretical perspective for examining the disclosure patterns of participants of DAC. Our study revealed four key findings pertaining to: support for disclosure about DAC to donor conceived people and significant others; overall high levels of attachment security in participants of DAC; greater attachment insecurities, particularly *Avoidance*, associated with disclosure to fewer individuals in nominated social networks; and the influence of *Avoidance* attachment reducing perceived relational support, which may have bearing upon longitudinal disclosure decisions.

### Attitude to disclosure about DAC

Knowledge about one’s genetic heritage is considered integral to self-identity and more so where genetic lineage deviates from expected familial blood lines (Turner and Coyle, 2000; Mahlstedt *et al.*, 2010; Slutsky *et al.*, 2016). Consequently, transparency with donor conceived children about circumstances of conception is promoted by health professionals in many western countries, including Australia, as the model of ‘best practice’ (Hammarberg *et al.*, 2011; Australian and New Zealand Infertility Counsellors’ Association (ANZICA), 2018). Our study shows that the majority of respondents, irrespective of whether they were recipients or donors, were supportive of disclosure to a person born following DAC about the circumstances of their conception.

Despite participants’ expressing a perspective that supports the importance of disclosure, this sentiment may not always translate into actual disclosure about one’s involvement with DAC. In this study, irrespective of family structure or donor programme type, the majority of recipients and donors had already disclosed their participation in a DAC programme to their child(ren), with age of child being the primary disclosure barrier. In the context of family dynamics, communication, and family functioning, this finding is reassuring as the burden of secrecy and non-disclosure about infertility and DAC can be associated with increases in conflict and negative emotional sequelae (Paul and Berger, 2007; Slade *et al.*, 2007; Shehab *et al.*, 2008; Daniels *et al.*, 2011; Frith *et al.*, 2018).

Studies suggest that women are more open to disclosure about family formation processes and thus are more likely to reveal to others their participation in a fertility programme than men (Schmidt, 2009). Although proportionally fewer men were involved in this study, there were no differences between sperm and oocyte donors in their disclosure practices. Overall, the majority of respondents, irrespective of gender or programme type had disclosed to someone about their involvement in DAC, with few respondents (3.8% recipients; 5.4% donors) keeping their involvement in DAC a total secret. These findings differ from the historical donor conception literature where non-disclosure attitudes prevailed particularly amongst partnered heterosexual recipients of donor insemination (Daniels, 1987; Brewaeys *et al.*, 1997; Berger and Paul, 2008; Bay *et al.*, 2014); although are commensurate with shifts in legal frameworks mandating donor programmes using open-identity donors found in Australia and other international jurisdictions (Blyth and Frith, 2009; Daniels *et al.*, 2011; Hammarberg *et al.*, 2011; Allan, 2012; Dempsey *et al.*, 2019; Ishii and De Miguel Beriain, 2022).

### Attachment style, DAC, and disclosure patterns

Bowlby *et al.* have suggested that an individual’s attachment style could shape their psychological and physiological adjustment towards experiences appraised as stressful (Bowlby, 1969; Bowlby, 1988; Mikulincer and Florian, 1995; Shaver and Mikulincer, 2007). Therefore, attachment insecurities (*Anxiety* or *Avoidance*) may be critical to an individual’s confidence in confiding in others about personal matters (Bowlby, 1969; Mikulincer and Nachshon, 1991; Slepian and Kirby, 2018). Interestingly, participants in this study reported overall lower scores on attachment *Anxiety* and *Avoidance* when compared to the study instrument (ECR-RS) reference group (Fraley *et al.*, 2011) and to a community based Australian adult sample (Christian *et al.*, 2019). This finding suggests a more secure attachment style and may reflect the characteristics of individuals who accept alternative, non-traditional pathways to family formation. Future research could draw on qualitative data to explore this proposition further.

Whilst both forms of attachment insecurities have bearing on disclosure practices, our analyses highlighted a stronger association between higher attachment *Avoidance* scores and reduced disclosure of participation in a DAC programme to individuals’ wider social network (i.e. to close family and friends). This finding is in line with attachment principles as elevated attachment-related insecurities, particularly the *Avoidance* dimension, can result in a reluctance in self-disclosure, potentially owing to perceived negative views by others (Slepian and Kirby, 2018; Jaffe and Douneva, 2020). This can include perceptions of defectiveness, shame, and rejection placing that individual at risk of emotional distance on matters of importance, particularly during stressful situations (Mikulincer *et al.*, 2003; Wei *et al.*, 2005; Shaver and Mikulincer, 2007; Mikulincer and Shaver, 2016). From a clinical perspective, this finding underscores the need for counsellors to understand the implications of attachment theory and assess for attachment insecurities during pre-treatment implications counselling. Importantly, as a target of clinical intervention, counsellors can provide psychoeducational strategies to help challenge clients’ negative patterns of interpersonal interactions (e.g. secrecy, fears of rejection, and emotional cut-off) and foster support networks that would be in the best interest of the child and family.

### Support network, attachment, and disclosure

Inherently, maintaining and sharing secrets can influence how close someone feels towards another (Jaffe and Douneva, 2020) with topic avoidance, that is non-disclosure about important information, potentially impinging upon psychological wellbeing and relationship satisfaction (Caughlin and Afifi, 2004; Afifi and Caughlin, 2006). Both partner and social support are known to improve psychological adaptation and transition to parenthood. Relationship support can be particularly important in the context of family formation involving ART where the emotional and physical burden can increase (Hammarberg *et al.*, 2008; Martins *et al.*, 2014). Overall, our findings showed that a higher score on the *Avoidance* attachment domain was associated with lower levels of perceived social support, albeit level of perceived support was generally quite high in this study cohort. It is possible that support scores in our study were positively skewed to maintain a positive impression, although they are in line with other studies that suggest individuals accessing fertility treatment view their support, particularly romantic partner support, as paramount in mitigating the burden of fertility treatment (Martins *et al.*, 2014). Moreover, this finding to some extent may arguably be a reflection of the Australian mandated pre-treatment implications counselling process (Australian and New Zealand Infertility Counsellors’ Association (ANZICA), 2018) that explores relationship dynamics and communication prior to commencement of treatment, such that only emotionally in tune couples actually proceed with DAC.

Previous research has found that an individual’s attachment to their parental figures can be divergent and hence dyad specific (Howes and Spieker, 2008); a finding that was replicated in part in this study. In this study, recipients and donors who had not fully disclosed to their parents had significantly higher scores on *Avoidance* attachment to that particular parent. This occurred irrespective of the presence of strong partner support or broader social network. For recipients, higher relationship-specific *Avoidance* scores primarily influenced rates of disclosure patterns with their mother or father. Amongst donors, those with higher scores on *Anxiety* or *Avoidance* were less likely to have fully disclosed to their father. Interestingly, a greater percentage of recipients and donors had disclosed to close friends over their father or in-laws. It is possible that close friends may similarly be at the life stage of family formation and thus represent a safe base for discussion. Moreover, topic avoidance in family situations can arise where there may be deviations from social norms (e.g. traditional family formation) and where risks of reactions/familial rejection have the potential to raise greater distress and thus reduce communication (Slade *et al.*, 2007; Slepian and Kirby, 2018; Jaffe and Douneva, 2020). Future in depth qualitative studies that explore understanding of these disclosure differences based on attachment principles would be helpful in informing implications counselling and psychotherapeutic practice relevant to participants of DAC. Finally, our results showed that proportionally more single women had disclosed the full circumstances of their fertility treatment than either partnered heterosexual or same sex couples. The latter finding was surprising as it would have been anticipated that same sex couples may have had equivalent or higher levels of disclosure to their social network given their sexual orientation transparently pre-empts the need for DAC. As the study was anonymously completed online survey, an opportunity to tease out this finding was not possible, although we speculate that the desire to safeguard perceptions of equal parental status and enhance family bonding irrespective of genetic links (Nordqvist, 2010; Raes *et al.*, 2014; Łukasiewicz and Allan, 2022) may at times result in partnered recipients, either heterosexual or same sex, non-disclosing specific donor programme details to others (e.g. articulating whether the donor was known or unknown). Future studies should explore in greater depth the implications of disclosure from the partner perspective since they too have a key role to provide in supporting transparent disclosure about DAC with confidence.

### Strengths and limitations

The results of our study need to be considered in the context of the following methodological limitations. First, exploring whether there is an association between insecure attachment-related dimensions and disclosure about DAC through a cross-sectional survey does not necessarily confirm causality or provide unequivocal assumptions about disclosure longitudinally. Second, no information is available on the characteristics of participants who decided not to participate, with the potential of respondent bias operating. That is, it is possible that only those participants who were in securely attached, supportive relationships felt confident to participate in a study on disclosure practices. Third, the reported disclosure patterns may be owing to utilization of the mandated implications counselling provided by ANZICA (https://www.fertilitysociety.com.au/professional-groups-anzica-australia-new-zealand/) required in Australia, rather than attachment processes *per se*. Future studies exploring attachment theory and disclosure in jurisdictions whereby counselling is not mandated prior to commencing fertility treatment may assist in disentangling this factor. Notwithstanding the aforementioned limitations, the strengths of the present study include the use of a large, representative, fertility cohort drawn from multiple ART clinics and patient forums, thereby involving individuals where current and prospective matters of disclosure are important. In addition, the exploration of gamete/embryo donors’ disclosure practices to their own children and significant others provides new understanding relevant to this field of research (Crawshaw and Daniels, 2019). Finally, while the concept of attachment theory has been applied to some areas of fertility research, its conceptualization as a framework for considering disclosure practices is novel and thus a key strength of this study. Future studies exploring donor disclosure practices in the context of attachment theory across different cultures and different types of ART treatment procedures would thus have merit.

### Clinical implications

The increasing use of open-identity donor-assisted treatment programmes in the era of readily available DNA tests, online genealogy websites, and emerging digital technology has meant that the notion of absolute donor anonymity no longer applies (Hertz and Mattes, 2011; Harper *et al.*, 2016; Crawshaw, 2018; Pennings, 2019; Ishii and De Miguel Beriain, 2022; Łukasiewicz and Allan, 2022). Therefore, in order to better prepare prospective individuals and couples involved in the donor conception process, infertility counsellors could consider the merits of assessing attachment-related anxiety and avoidance dimensions during pre-treatment implications counselling as a framework of understanding a patient’s conceptual model of interpersonal communication, maintaining secrets and thus risks of non-disclosure (Afifi and Caughlin, 2006; Paul and Berger, 2007). Relatedly, parental attachment security may have a positive influence on children’s thoughts and feelings about their donor (Zadeh *et al.*, 2017). Thus, information about attachment could then be used to develop targeted interventions aimed at improving knowledge and confidence in disclosure practices to optimize future DAC stakeholder contact experiences.

## Conclusion

Family formation with the assistance of a donor is on the rise and, with changing legal frameworks moving towards open-identity donor programmes, historically held views regarding concealment of donor practices have changed. This means that disclosure to significant others about involvement in DAC is relevant to explore, particularly if family held views about biological identity and family genetic connectedness are deemed important. Attachment theory is built on the foundation of strength of interpersonal relationships as a means to seek support and regulate emotions during times of distress. Placing disclosure practices within an attachment framework offers a novel explanation for why hesitancy about disclosure may occur. This study highlights that *Avoidance* attachment influences full disclosure about involvement in DAC to significant family and friends. Finally, the findings highlight the clinical utility for the introduction of attachment screening during donor implications counselling that may assist with donor conceived identity needs, and potentially improve relationship outcomes based on transparency in DAC arrangements.

## Data Availability

The data underlying this article cannot be shared publicly for the privacy reasons of the individuals that participated in the study. Participants did not consent for their data to be released.
